# A correlation analysis of sacrococcygeal chordoma imaging and clinical characteristics with the prognostic factors

**DOI:** 10.3389/fonc.2022.1012918

**Published:** 2022-09-26

**Authors:** Fei Zhao, Shujian Tian, Lei Zheng, Yue Li, Lu Zhang, Song Gao

**Affiliations:** ^1^ Department of Orthopedics, Henan Provincial People’s Hospital, People’s Hospital of Zhengzhou University, Zhengzhou, China; ^2^ Department of Radiology, Henan Provincial People’s Hospital, People’s Hospital of Zhengzhou University, Zhengzhou, China

**Keywords:** Chordoma, imaging, recurrent, prognosis, survival analysis

## Abstract

**Objective:**

To investigate the imaging and clinical risk factors related to the postoperative recurrence of sacrococcygeal chordoma.

**Methods:**

63 patients of sacrococcygeal chordoma proved by operation and pathology in our hospital from January 2009 to December 2019 were retrospectively analyzed in the related factors of imaging manifestations, pathological type, and extent of surgical resection. The recurrence of sacrococcygeal chordoma was followed up. Univariate Kaplan-Meier survival analysis and multivariate Cox regression analysis were used to analyze the related factors of recurrence.

**Results:**

On plain radiographs and CT scans, chordoma primarily manifested as osteolytic bone loss and uneven soft tissue mass, with typical calcification or ossification (56.1 percent). Numerous chunk nodules with clearly high signal levels and short signal intervals were seen as the “pebble” in MRI characteristics on T2WI. The follow-up period ranged from 20 to 130 months, with a median time of 47.5 months. There were 14 recurrences (22. 2%) during the follow-up period. 13 patients with recurrence underwent surgery again, and 5 of them recurred after surgery (recurrence time range 3 to 97 months, median 38. 5 months). 6 (42.8%), 8 (57. 1%), and 13 (92. 9%) of the 14 patients with recurrence recurred within 2, 3, and 5 years after surgery, respectively. Univariate Kaplan-Meier survival analysis showed that occurred with local infiltration, Low differentiated chordoma, partial resection had a high postoperative recurrence rate, and all differences were statistically significant (P<0.05). Multi-factor Cox regression analysis showed whether local infiltration occurred and the degree of tumor resection were independent risk factors for tumor recurrence.

**Conclusion:**

Sacrococcygeal chordoma has a high tendency of recurrence, and the likelihood of recurrence is higher in tumor occurred with local infiltration, non-complete tumor resection and low differentiated chordoma, which can be considered to shorten the review cycle and complete tumor resection as much as possible during surgery.

## Introduction

Chordoma is a low-grade malignant bone tumor that develops from the residual tissue of the chordal bone of the mid-axis and accounts for about 3% to 4% of all primary malignant bone tumors (1). The tumor grows slowly but has a high level of local aggressiveness (2). Due to a lack of specificity in clinical symptoms and imaging, up to 70% of patients with chordoma are misdiagnosed or missed (3). As a result, fully comprehending the imaging signs of chordoma is critical for early diagnosis and complete surgical resection. According to previous reports (4), the local recurrence rate after surgical resection of sacral chordoma is high, up to 43%-85%, and the recurrence usually occurs within 3 years after surgery. Postoperative recurrence and multiple operations can seriously affect the quality of life of patients, and patients with chordoma may survive for a long time, so local control is more important. It is also worthwhile to study and analyze the risk factors that may affect postoperative recurrence. Therefore, this study retrospectively analyzed the data of sacral chordoma cases undergoing surgical treatment in our hospital, evaluated and analyzed the imaging manifestations and related factors affecting postoperative recurrence, and provided referential data and relevant experience for clinical work and research.

## Data and methods

### Patients

A total of 63 patients with chordoma diagnosed by pathology after surgical treatment in Henan Provincial People’s Hospital from January 2009 to December 2019 were collected. Inclusion criteria: ① all patients were pathologically confirmed as sacrococcygeal chordoma; ② preoperative imaging (CT or MRI) was performed; ③ without any treatment before examination. ④ Postoperative clinical and follow-up data were complete. Exclusion criteria: ① radiotherapy was administered before surgery; ② pathological specimens originated from puncture biopsy rather than surgical resection. ③ Patients who were lost to follow-up and died of other diseases during follow-up.

Adjuvant local postoperative radiotherapy was performed for patients with definite residual tumor tissue and incomplete resection during operation. Follow-up methods: The patients were reexamined every 3 months for 1 to 2 years after operation, every 6 months for 3 to 5 years after operation, and once a year thereafter. The follow-up deadline is July 2022. The reexamination included sacrococcygeal X-ray, local B-ultrasound, sacrococcygeal CT or MRI, chest CT, and whole-body bone SPECT scan. Recurrence was defined as (1): readmission due to tumor recurrence, which was confirmed by postoperative pathology (2); MRI or CT imaging examination showed that the lesion tissue reappeared in the original surgical site in the patients with total resection, or the residual lesion tissue in the patients with partial resection was larger than before. Recurrence free survival (RFS) was calculated, which was the time from the first operation to the recurrence of the lesion. Patients who were lost to follow-up or had no recurrence at the end of follow-up were used as ending values.

### Observed indicators

For patients with sacrococcygeal chordoma, the age, gender, tumor location (with the level of sacral 3 as the boundary), maximum tumor diameter, local infiltration, intratumoral calcification, pathological type, and extent of surgical resection were collected. The criteria for local infiltration are: the tumor is poorly demarcated from adjacent tissues and involves adjacent muscles, fatty spaces or other soft tissue in or around the spinal canal. The criteria for intra-tumoral calcification are: patchy, speckled high density in the tumor on CT or low signal in T1WI and T2WI inside the tumor on MRI. Pathological typing: According to the typing method proposed by Carstens in 1995, there were three subtypes: conventional (classic) chordoma, chondroid chordoma, and low differentiated (actively growing) chordoma. The surgical boundaries were evaluated according to the surgical staging of tumors of the musculoskeletal system (Enneking stage), which was divided into extensive resection (resection distance of 1.5 cm or more from the tumor margin), marginal resection, and intracapsular resection.

### Statistical methods

SPSS19.0 statistical software was used for statistical analysis of the data. The Kaplan-Meier method was used for survival analysis to compare the effect of gender, tumor location, pathological type, local infiltration, intratumoral calcification, and the extent of surgical resection on recurrence-free survival time, and the Log-rank univariate hypothesis test was performed. Age and maximum tumor diameter were compared by t-test. Multivariate Cox regression analysis was performed for P<0.05 in univariate analysis.

## Results

### Clinical data and imaging findings

**Figure 1:**
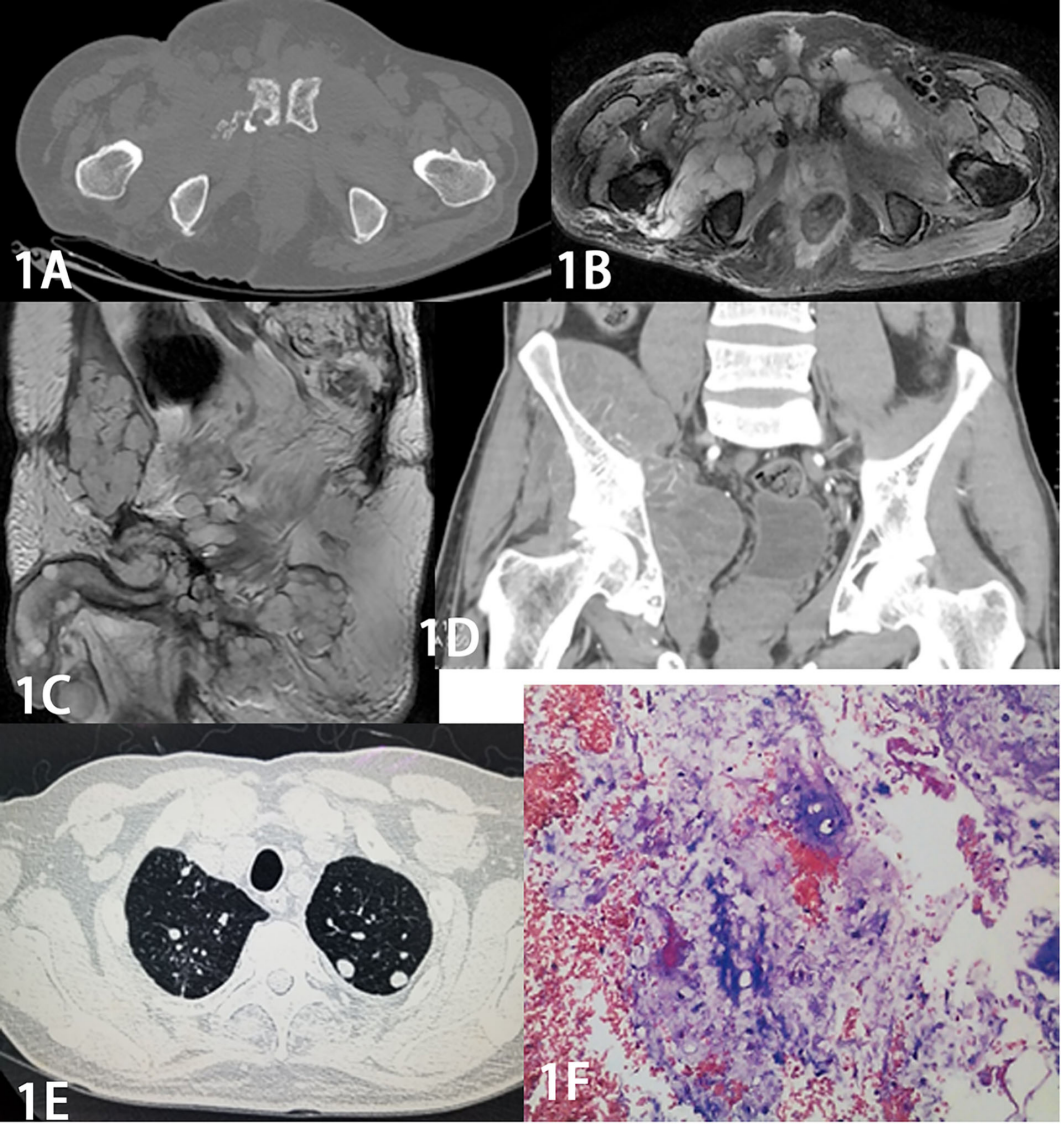
Male, 64 years old, with a former sacrococcygeal chordoma that recurred 8 times over 9 years. The recurrent tumor increased significantly in extent with the number of recurrences. Figure 1A Axial CT shows osteolytic destruction of the right side of the pubic symphysis with a soft tissue mass. Figure 1B MR axial and Figure 1C sagittal T_2_ WI pelvic area and bilateral medial thigh masses showed “pebble-like” clusters of apparently high-signal mass nodules with low-signal intervals. Figure 1D Coronal CT enhancement scan shows significant inhomogeneous enhancement. Figure 1E CT shows multiple small metastatic nodules in both lungs. The pathology of Figure 1F shows a few small round cells scattered in a background of mucus-like stroma, and combined with the immunohistochemical results (S-100 protein positive, GFAP positive, D2-40 positive, EMA negative), the diagnosis of chordoma was made.

A total of 63 patients with primary sacrococcygeal chordoma were collected, including 53 males and 10 females. The age range was 25 to 83 years, with a median age of 55.5 years. Tumor location (bounded by the level of sacral 3): 19 cases above sacral 3 and 44 cases below sacral 3. The mean maximum tumor diameter was 9.1 cm (range 4.0-16.7 cm) measured by preoperative CT and MRI. CT examination showed distended osteolytic bone destruction of the affected sacrum or coccyx, with soft tissue masses of uneven density around them. In 23 cases, irregularly scattered calcification or ossification shadows were seen in the lesion. The masses were characterized by “pebble-like” clusters of distinct T2WI high-signal nodules with low-signal intervals on MRI. 22 cases were combined with local infiltrates that were poorly demarcated from adjacent muscles, dural sacs, or posterior soft tissues of the vertebral body (**Figure 1**). The extent of surgical resection: intracapsular resection was performed in 24 cases, marginal resection in 26 cases and extensive resection in 13 cases.

### Recurrence

**Figure 2:**
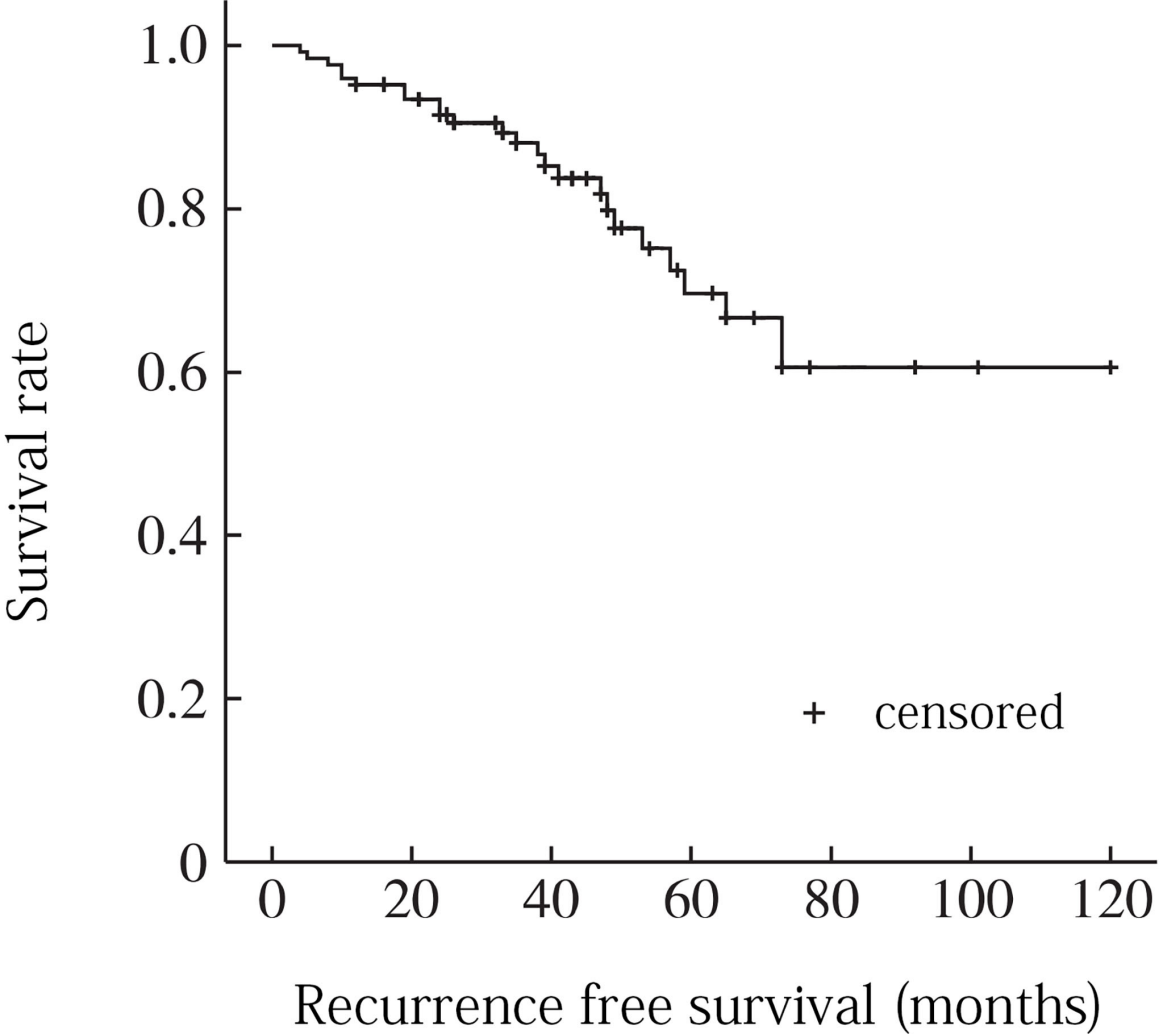
Overall recurrence-free survival curve for sacrococcygeal chordoma.

The follow-up period ranged from 20 to 130 months, with a median time of 47.5 months. During the follow-up period, 14 patients (22. 2%) recurred. 13 patients with recurrence underwent surgery again, and 5 of them recurred after surgery (recurrence time range 3 to 97 months, median 38. 5 months). 6 (42. 8%), 8 (57. 1%), and 13 (92. 9%) of the 14 patients with recurrence recurred within 2, 3, and 5 years after surgery, respectively. The Kaplan-Meier analysis showed recurrence-free survival rates of 86.7% and 67.5% at 3 and 5 years, respectively (**Figure 2**).

### Analysis of factors affecting recurrence

**Figure 3:**
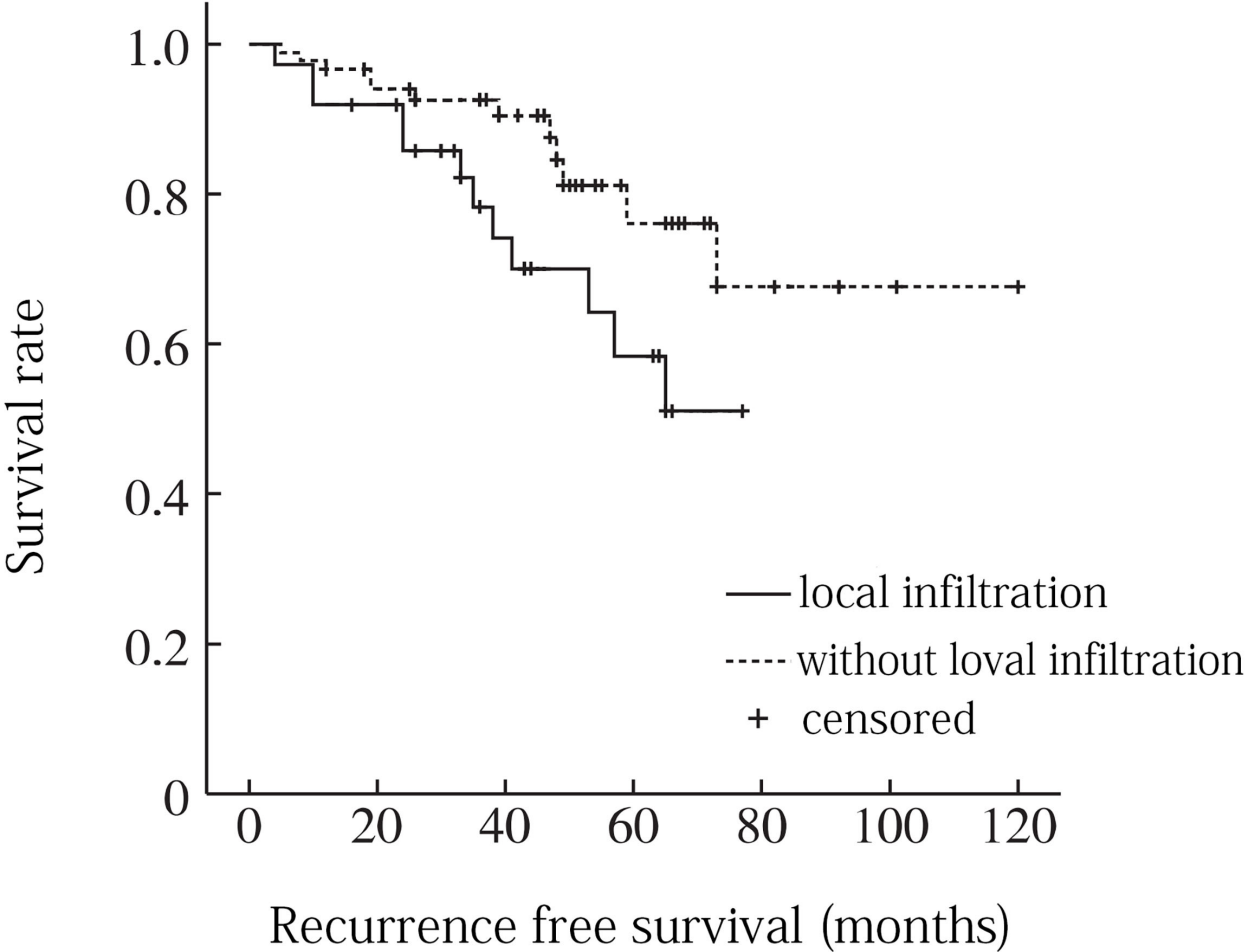
Recurrence free survival curve for sacrococcygeal chordoma with or without local infiltration .

**Figure 4:**
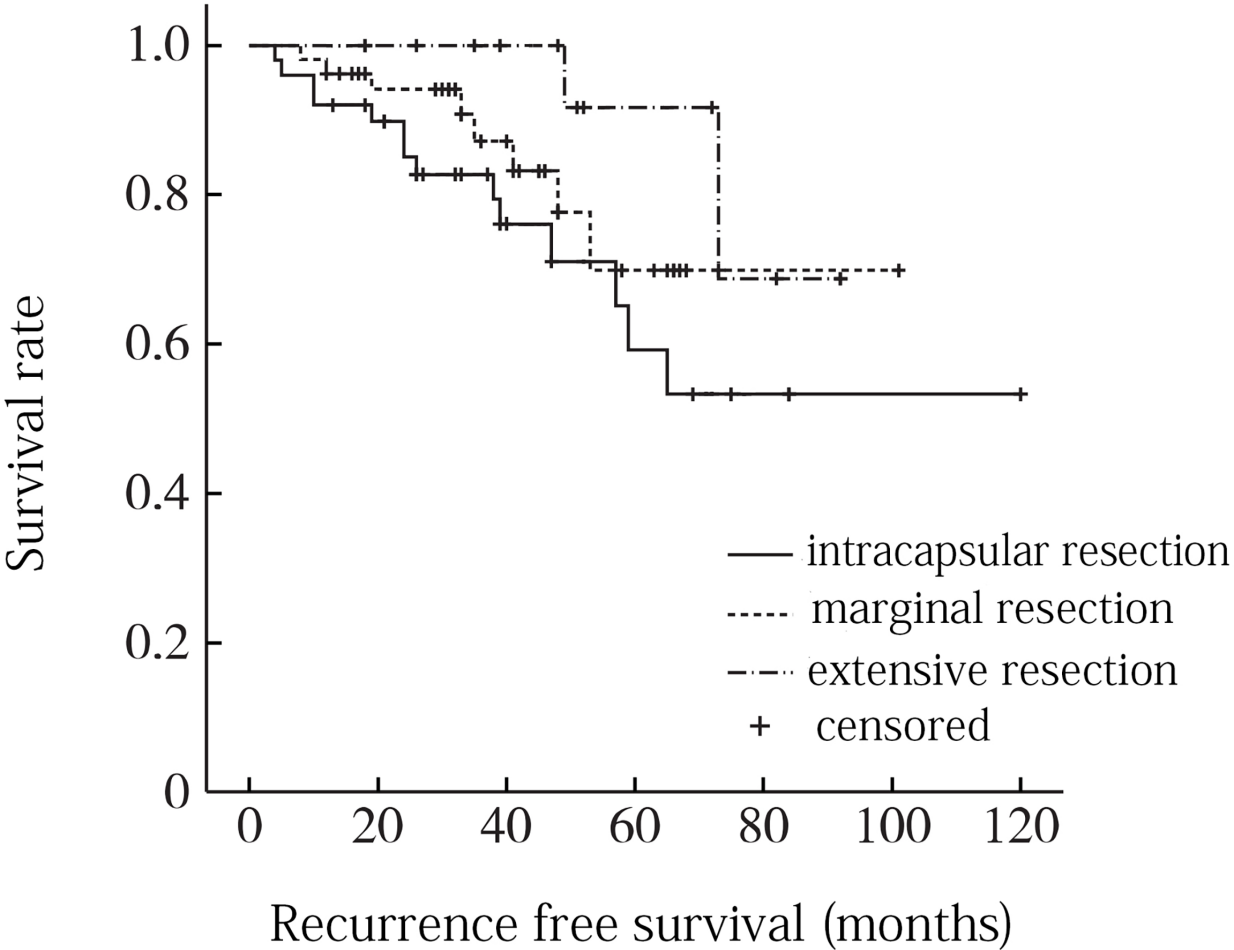
Recurrence free survival curve for different surgical resection ranges of sacrococcygeal chordoma.

A total of 8 factors including age, gender, tumor location, tumor size, local infiltration, intratumoral calcification, pathological type and surgical resection extent were included in univariate analysis. Age factor analysis: the average age of recurrence group was 52.8 years, and the average age of non-recurrence group was 56.5 years, and the difference was not statistically significant (P = 0. 116). Gender factor analysis: the recurrence rate of male was 22.6% (12/53), which was higher than that of female 20. 0% (2/10), and the difference was not statistically significant (P = 0. 201). Analysis of tumor location factors: the recurrence rate above sacral 3 was 26.3% (5/19) compared to 20.5% (9/44) below sacral 3, with no statistically significant difference (P = 0. 151). Analysis of tumor size factors: the average maximum diameter of tumor in recurrence group was 9.2 cm, and that in non-recurrence group was 8.8 cm, and the difference was not statistically significant (P = 0. 352). Analysis of local infiltration: the recurrence rate of local infiltration was 29.0% (9/31), which was higher than 15.6% (5/32) without local infiltration, and the difference was statistically significant (P = 0.001). Factors analysis of intratumoral calcification: the recurrence rate of intratumoral calcification was 21.7% (5/23), which was lower than that of non-intratumoral calcification (22.5%, 9/40), and the difference was not statistically significant (P = 0. 683). Analysis of pathological type: the recurrence rates of conventional chordoma, chondroid chordoma, and low differentiated chordoma were 22.2% (10/45), 15.4% (2/13) and 40.0% (2/5), respectively (P =0. 045). Analysis of surgical resection extent: the recurrence rates of intracapsular resection, marginal resection and extensive resection were 31.2% (8/24), 21.1% (5/26) and 7.7% (1/13), respectively, and the differences were statistically significant (P = 0.009) (**Table 1**). When the statistically significant indicators in univariate analysis were included in multivariate analysis, local infiltration (P = 0. 035) and surgical resection range (P = 0. 005) were independent risk factors for recurrence (**Figures 3**, **4**).

**Table 1:** Results of a univariate Kaplan-Meier survival analysis affecting postoperative recurrence of sacrococcygeal chordoma.

	Number of cases	Number of recurrences (%)	*P* value
**Sex**			0.201
Male	53	12(22.6%)	
Female	10	2(20.0%)	
**Tumor location**			0.151
Above sacral 3	19	5(26.3%)	
Below sacral 3	44	9(20.5%)	
**Local infiltration**			0.001
Yes	31	9(29.0%)	
No	32	5(15.6%)	
**Intratumoral calcification**			0.683
Yes	23	5(21.7%)	
No	40	9 (22.5%)	
**Type of pathology**			0.045
Conventional	45	10(22.2%)	
Chondroid	13	2(15.4%)	
Low differentiated	5	2(40.0%)	
**Extent of resection**			0.009
Intracapsular resection	24	8(31.2%)	
Marginal resection	26	5(21.1%)	
Extensive resection	13	1(7.7%)	

## Discussion

Chordoma is the most common primary malignant bone tumor after osteosarcoma, chondrosarcoma, and fibrosarcoma. It develops from residual or ectopic embryonic chordal tissue. It predominantly affects middle-aged and older adults over 40 years of age. Both sexes can be involved, and there is no difference in incidence. Many relevant clinical factors have been found to be associated with the prognosis of chordoma (5, 6). In this study, we analyzed possible associated risk factors by following 63 cases of sacrococcygeal chordoma after surgical treatment and found that the occurrence of local infiltration of the tumor and the border of surgical resection were independent risk factors for postoperative recurrence.

### Relationship between surgery-related conditions and prognosis

It is widely accepted that whenever possible, total resection should be pursued (7, 8). Chordoma in the sacrococcygeal region and other areas have a relatively high rate of total resection. At present, for chordoma, the first choice is a more complete surgical resection. Those with postoperative residual recurrence are then treated with adjuvant radiation therapy, and this treatment option is affirmed by the vast majority of domestic and international scholars (9). Surgical resection is the most important treatment for sacral chordoma. The margin of surgical resection is closely related to postoperative recurrence. This study showed that the postoperative recurrence rate of different surgical boundaries was statistically significant. For example, the recurrence rate of intracapsular resection was 31.2%, marginal resection was 21.1%, and extensive resection was only 7.7%. According to the principles of bone tumor surgery proposed by Enneking, the primary malignant bone tumor should at least be extensively excised. Marginal excision may leave the satellite foci of the tumor, while intracapsular excision may leave the tumor tissue directly, or even cause the tumor to spread around, leading to postoperative recurrence. However, due to the special location and anatomical structure of sacral chordoma, as well as the consideration of sacral nerve preservation and postoperative function, it is often difficult to reach the boundary of extensive surgical resection in clinical practice. According to multiple literature reports, the recurrence rate after extensive resection is 0-60%, while that after extensive resection is 43%-85% (10). Radaelli et al. (11) reported long-term follow-up results of sacral chordoma cases after surgical treatment, and 38 out of 99 cases had local recurrence. Surgical boundary was an independent risk factor for recurrence. Xie et al. (12) reported the treatment results of 54 cases of sacral chordoma, among which 41 cases (75.9%) did not reach the boundary of extensive resection, and the overall postoperative recurrence rate was 55.6% (30/54). Unsafe surgical resection margin is a significant factor leading to postoperative recurrence.

### Relationship between imaging signs and prognosis

CT combined with MRI can help assess the location, size, borders, internal tumor components, and marginal infiltration of the tumor (13). The effect of tumor size on patient recurrence or metastasis after surgery has been reported differently. Larger tumor size has a negative impact on both progression-free survival and overall survival (14). However, the results of our analysis showed that tumor size was not an independent predictor of recurrence. This may be due to the limited distribution of tumor size and the small sample size of our study population, which was only hospitalized patients. Chordoma often has scattered calcifications within the tumor, which are more sensitive on CT, and double low signal shadow on MRI as T1WI and T2WI. In this study, we found that the presence or absence of scattered calcifications within the tumor did not significantly affect the time to recurrence or metastasis after chordoma surgery. Therefore, this factor of intra-tumor calcification can only be used as a diagnostic imaging basis for chordoma and not as an indicator of prognosis. Chordoma has a high degree of local invasiveness, and MRI is more sensitive and clearer in showing the borders of the tumor. Patients with local infiltration have a significantly higher rate of recurrence or metastasis (15). The rate of recurrence is significantly higher in patients with local infiltration. Because the local anatomy of the spine is complex and rich in blood vessels, it is difficult to reveal the tumor during surgery, and if local infiltration occurs, it is often difficult to remove the tumor completely (16).

### Relationship between pathological findings and prognosis

Zou M X et al. (17) identified the type of pathology as a relevant factor affecting the postoperative prognosis of chordoma in their study. Chondroid chordoma is characterized microscopically by a large amount of mucinous stroma with lobular structures separated by lacunar fibrous septa and containing more or less hyaline chondrocyte-like areas in addition to more mucin-rich vacuolated cells (18). The age of onset of this tumor is young and it is easily confused with chondrosarcoma, but its prognosis is better than that of conventional chordoma and chondrosarcoma. Numerous immunohistochemical studies have also found that chondroid chordoma and conventional chordoma were positive for the epithelial marker antigen cytokeratin, whereas chondrosarcoma is negative, so chondroid chordoma is indeed a specific type of chordoma. The 13 cases of chondroid chordoma in our study were significantly less likely to have recurrence after surgery than the conventional or low differentiated types in univariate analysis (P=0.045). In addition to pathological types, some immunohistochemical indicators are also closely related to the prognosis of chordoma. The proliferative viability of the tissue can be measured by applying the MIB-1 index, a monoclonal antibody against Ki-67 antigen. Recent literature has shown that MIB-1 index increases with tumor recurrence and can be used to measure the risk of recurrence in chordoma (19, 20). Moreover, some studies have suggested that chordomas with high MIB-1 index and low expression of E-cadherin are expected to have an aggressive course and to be associated with a high recurrence rate despite macroscopic total extirpation during surgery. Patients whose tumors have high MIB-1 index and low E-cadherin expressions should be followed meticulously (21).

### Research prospects: Potential molecular markers associated with chordoma prognosis

With advances in various treatments, the rate of local control of chordoma has been effectively improved. However, the recurrence rate of chordoma remains high, and the efficacy of re-surgery or re-radiotherapy for patients with recurrence is very limited. Therefore, as a new direction in tumor treatment, molecular targeted drug therapy has been increasingly investigated by scholars. The vitro studies conducted using chordoma cell lines have suggested that brachyury plays an important role in chordoma progression (22). Moreover, some other relevant studies suggest that high brachyury expression and T gene CNG are associated with the risk of recurrence or shortening of Progression-free survival in skull base chordomas. The brachyury-negative chordomas are biologically distinct from brachyury-positive chordomas and that T/brachyury might be an appropriate molecular target for therapy of these neoplasms (23). In a recent study, Otani et al. also suggested that activation of the PI3K/Akt pathway also upregulated Brachyury expression and promoted chordoma cell growth *in vitro*. Brachyury, or molecules involved in PI3K/Akt pathway activation upstream of Brachyury, may represent important new targets for chordoma treatment (24). Overall, their findings could be potential therapeutic molecular targets and used to develop a potential promising therapeutic strategy for the treatment of sacrum chordoma (25).

### Shortcomings of this study

① The number of cases was not large enough, as our hospital saw far more than 63 patients with sacrococcygeal chordoma from January 2009 to December 2019, but some of the cases were recurrences after external surgery and could not be included in the study; ② The overall incidence of sacrococcygeal chordoma was low, and in order to summarize the data of a larger sample of cases, the consultation period in this study spanned a long period of time, and the examination and treatment options differed between time periods. ③ Some more detailed analyses did not include pathological markers like MIB-1 and molecular markers like brachyury and PI3K/AKT/mTOR, which could lead to novel therapies and improve chordoma prognosis. The expectation is that these factors will be included in our subsequent studies of prognostic correlates of chordoma.

## Conclusion

In conclusion, sacrococcygeal chordoma is a disease with a high risk of recurrence. Understanding its typical imaging features is helpful for early diagnosis, preoperative and postoperative evaluation of the disease. Through the treatment and follow-up of this group of cases, we found that local invasion and surgical resection margin were significant factors affecting postoperative recurrence. Therefore, early imaging diagnosis and auxiliary judgment of invasion margin, good local control and reduction of postoperative recurrence rate are still important issues to be solved.

## Data availability statement

The original contributions presented in the study are included in the article/supplementary material. Further inquiries can be directed to the corresponding author.

## Ethics statement

The studies involving human participants were reviewed and approved by Medical Ethics Committee of Henan Provincial People’s Hospital. The patients/participants provided their written informed consent to participate in this study.

## Author contributions

SG was the general responsible person of the project, responsible for the design of the project, writing and checking the article, and responsible for the reliability of the article. ST formulated the overarching research goals and aims. FZ was responsible for the design of the experiment, the analysis of the results, and the writing of the main part of the manuscript. FZ also participated in the development of the whole experiment, planned the main parts of the experiment, and analyzed the results. SG and ST were also responsible for the coordination and review of this study. YL participated in the planning and execution of the experiment, and assisted in the specific analysis and verification of patients’ images. LuZ was responsible for patient enrollment, imaging and clinical information collection, and data collation. All authors read and approved the final manuscript.

## Conflict of interest

The authors declare that the research was conducted in the absence of any commercial or financial relationships that could be construed as a potential conflict of interest.

## Publisher’s note

All claims expressed in this article are solely those of the authors and do not necessarily represent those of their affiliated organizations, or those of the publisher, the editors and the reviewers. Any product that may be evaluated in this article, or claim that may be made by its manufacturer, is not guaranteed or endorsed by the publisher.
